# dAcquisition setting optimization and quantitative imaging for ^124^I studies with the Inveon microPET-CT system

**DOI:** 10.1186/2191-219X-2-7

**Published:** 2012-02-13

**Authors:** Nadège Anizan, Thomas Carlier, Cecilia Hindorf, Jacques Barbet, Manuel Bardiès

**Affiliations:** 1 Institut National de la Santé et de la Recherche Médicale (INSERM) U-892, Nantes, 44035, France; 2 Department of Medical Imaging, ONIRIS, Nantes, 44307, France; 3 Institut National de la Santé et de la Recherche Médicale (INSERM), Toulouse, 31062, France

**Keywords:** small animal imaging, PET/CT, iodine-124, quantitative imaging

## Abstract

**Background:**

Noninvasive multimodality imaging is essential for preclinical evaluation of the biodistribution and pharmacokinetics of radionuclide therapy and for monitoring tumor response. Imaging with nonstandard positron-emission tomography [PET] isotopes such as ^124^I is promising in that context but requires accurate activity quantification. The decay scheme of ^124^I implies an optimization of both acquisition settings and correction processing. The PET scanner investigated in this study was the Inveon PET/CT system dedicated to small animal imaging.

**Methods:**

The noise equivalent count rate [NECR], the scatter fraction [SF], and the gamma-prompt fraction [GF] were used to determine the best acquisition parameters for mouse- and rat-sized phantoms filled with ^124^I. An image-quality phantom as specified by the National Electrical Manufacturers Association NU 4-2008 protocol was acquired and reconstructed with two-dimensional filtered back projection, 2D ordered-subset expectation maximization [2DOSEM], and 3DOSEM with maximum *a posteriori *[3DOSEM/MAP] algorithms, with and without attenuation correction, scatter correction, and gamma-prompt correction (weighted uniform distribution subtraction).

**Results:**

Optimal energy windows were established for the rat phantom (390 to 550 keV) and the mouse phantom (400 to 590 keV) by combining the NECR, SF, and GF results. The coincidence time window had no significant impact regarding the NECR curve variation. Activity concentration of ^124^I measured in the uniform region of an image-quality phantom was underestimated by 9.9% for the 3DOSEM/MAP algorithm with attenuation and scatter corrections, and by 23% with the gamma-prompt correction. Attenuation, scatter, and gamma-prompt corrections decreased the residual signal in the cold insert.

**Conclusions:**

The optimal energy windows were chosen with the NECR, SF, and GF evaluation. Nevertheless, an image quality and an activity quantification assessment were required to establish the most suitable reconstruction algorithm and corrections for ^124^I small animal imaging.

## Background

Small animal imaging is an active area of research for the investigation of new pharmaceuticals and treatment regimens. *In vivo *imaging permits longitudinal studies to be performed [1]. Quantitative imaging also provides a basis for the calculation of the absorbed dose for radioimmunotherapy applications since it yields both the pharmacokinetics (SPECT or positron-emission tomography [PET]) and the anatomy and density of the tissues (computed tomography [CT]) [2]. Most PET studies are performed with ^18^F-labeled tracers. However, the short physical half-life (109.8 min) of ^18^F limits the study of pharmacokinetics that span several days, as can be the case for large molecules like monoclonal antibodies. Consequently, 'long-lived' β+ emitters, such as ^124^I (half-life of 4.17 days), have been proposed for both diagnostic and therapeutic studies [3]. However, the decay scheme of ^124^I limits image quality, as was established previously in clinical two-dimensional [2D] and three-dimensional [3D] PET imaging [4-6]. Roughly 12% of decays induce a positron emission followed in a few picoseconds by a 603-keV single photon. Another positron emission (≈11%) decays directly to the ground state, and approximately 28% of the remaining decays generate electron capture that decay mostly to the 603-keV level. In total, 63% of decays result in the emission of 603-keV single photons, while 10% produce 723-keV single photons for the two principal single-photon emissions [7]. Single photons emitted together with positrons may generate false coincidences (called gamma-prompt coincidences), and single photons emitted in cascade increase random coincidences that will contribute to the detection of a spurious background activity [8-10]. Therefore, it is necessary to optimize acquisition parameters to remove single photons while keeping annihilation photons. The aim of this work was to define optimal energy windows for ^124^I small animal imaging with the Inveon PET system (Siemens Medical Solutions USA Inc., Knoxville, TN, USA), based on the noise equivalent count rate [NECR]. The NECR represents the signal-to-noise ratio and is the most suitable parameter to determine the best compromise between true coincidences and undesirable events (random, scatter, and gamma-prompt coincidences). The optimal energy window was assessed for a mouse and a rat phantom to study the influence of the object size. Reconstruction algorithms and correction methods available in the manufacturer-supplied software (Inveon Acquisition Workplace 1.5) were then evaluated for ^124^I imaging with the National Electrical Manufacturers Association [NEMA] image-quality, mouse-sized phantom to assess the relative importance of each of them on image quantification. Imaging with ^18^F was also performed to demonstrate the effects of physical properties of ^124^I on image quality.

## Materials and methods

### The Inveon PET-CT system

The Inveon PET scanner is arranged as four detector rings of 16 blocks. Each block consists of a 20 × 20 lutetium oxyorthosilicate [LSO] crystal array of 1.5 × 1.5 × 10 mm^3 ^elements. The LSO blocks are optically coupled to Hamamatsu C-12 position-sensitive photomultiplier tubes (Hamamatsu Photonics, Iwata City, Japan). The 64 blocks are placed in a circular configuration with a diameter of 16.1 cm at the surface of the crystals, allowing for a transaxial field of view [FOV] of 10 cm and an axial FOV of 12.7 cm. Two lead rings with a 2.5-cm thickness are placed on either side of the detector rings to minimize the effects of activity outside the FOV. A list-mode file containing data on localization, time tag, and type of coincidence (prompt or delayed coincidence) is generated for each acquisition. Data can be sorted into 3D sinograms or directly into 2D sinograms by the single-slice rebinning [SSRB] algorithm. 3D sinograms can be then rebinned with the Fourier algorithm [FORE] or with the SSRB algorithm. Images can be reconstructed with 2D filtered back projection [2DFBP], 2D ordered-subset expectation maximization [2DOSEM], and 3DOSEM in combination with maximum *a posteriori *[3DOSEM/MAP]. Scatter correction is performed by single scatter simulation [SSS] correction, and the attenuation map is calculated from a CT transmission acquisition. All CT data considered in this work were acquired with a voltage set to 80 kV, a tube current of 500 μA, and a total rotation angle of 220° with 121 projections. CT data were reconstructed using FBP with the Shepp filter cutoff set to the Nyquist frequency.

### Count rate performance and scatter fraction

Count rate was evaluated using two polyethylene cylindrical phantoms as described in NEMA NU 4-2008 [11]. The mouse phantom had a diameter of 2.5 cm and a length of 7 cm. A hole with a 3.2-mm diameter was drilled parallel to the central axis at a radial offset of 1 cm. The rat phantom was manufactured in a similar geometry with a 5.0-cm diameter, a 15-cm length, and a hole at a 1.75-cm radial offset. The phantoms were filled with ^124^I and then with ^18^F. Activity injected in phantoms was measured with a dose calibrator (ACAD 2000, Lemer Pax, Carquefou, France) that was initially calibrated using a reference ^124^I source provided by IBA (Amsterdam, The Netherlands). The accuracy on the activity measurement was estimated to be better than 11% by combining uncertainties in the activity values (10%: value given by IBA) and in the dose calibrator measurements (5%: value given by the manufacturer). Data were acquired in a list-mode format then combined in 3D sinograms (with a span of 3 and a ring difference of 79) and converted into 2D sinograms by SSRB without normalization and with no correction for dead time, scatter, and attenuation. Prompt (true, scatter, and random coincidences) and delayed events were separated into two sets of data. The total (*R*_Total_), true (*R*_True_), random (*R*_Random_), scatter (*R*_Scatter_), and coincidence rates due to single photons (R_γ-Promt_) were calculated from the sinograms. Pixels located at more than 8 mm from the edges of the phantom were set to zero for the prompt- and delayed-event sinograms. The pixel with the largest number of counts was determined for each projection, and the projection was shifted to align this pixel with the central pixel of the sinograms. The aligned projections were summed for each slice. For the prompt sinogram frames, counts outside a 14-mm-wide strip centered on these profiles and under the line defined by linear interpolation between the edges of the 14-mm strip were assumed to represent the sum of scatter, random, and intrinsic coincidences due to intrinsic radioactivity of ^176^Lu in LSO crystals and coincidences due to single photons. The remaining counts were considered as true events. The random events were derived from the delayed-event sinograms. The gamma-prompt coincidence rate was estimated by a uniform distribution [12] and calculated as the mean of the 10 outermost projection bins on the prompt sinograms subtracted by delayed events (pixels located beyond 8 cm kept their value and were not set to zero). Acquisition of intrinsic radioactivity (R_intrinsic_) was performed for 10 h [13] (attenuation of the gamma-prompt coincidences and the intrinsic radioactivity due to the presence of the object size in the FOV were ignored).

The R_Scatter _was equal to:

(1) $$R_{\text{Scatter}}=R_{\text{Total}}-R_{\text{True}}-R_{\text{Random}}-R_{\gamma -\text{Prompt}}-R_{\text{intrinsic}}.$$

The scatter coincidence fraction [SF] was determined from the last acquisitions, in which the random rate was less than 1% of the true rate, and the true rate was five times that of the true intrinsic counts. The SF was calculated as:

(2) $$\text{SF}=\frac{R_{\text{Scatter}}}{R_{\text{Scatter}}+R_{\text{True}}}.$$

The gamma-prompt coincidence fraction [GF] was defined as:

(3) $$GF=\frac{R_{\gamma \text{-Prompt}}}{R_{\gamma \text{-Prompt}}+R_{\text{True}}}.$$

The NECR was determined according to Equation 4 from the prompt sinograms (without subtraction of delayed coincidences):

(4) $$\text{NECR}=\frac{{\left(R_{\text{True}}\right)}^{2}}{R_{\text{Total}}}.$$

The rat and mouse phantoms were filled with 4 MBq of ^124^I to determine the optimal energy window by changing the lower and upper level discriminators [LLD and ULD, respectively] [14]. For different LLD/ULD values, 1 × 10^6 ^prompt coincidences were recorded and NECR was calculated. Optimized LLD and ULD were defined at the maximum value of the NECR. The LLD was first fixed at 350 keV, and the ULD was increased in 10-keV steps starting at 530 keV. The optimal ULD was then fixed, and the LLD was decreased in 10-keV steps starting at 500 keV. Each phantom was then filled with 30 MBq of ^124^I, and the NECR curves were generated with optimized energy windows and with two coincidence time windows (2.8 and 4.7 ns). List-mode data were acquired every day for 15 days until activity in the phantoms decreased to a value of 1 MBq. The rationale for using the NECR figure of merit was to evaluate optimal acquisition settings (LLD, ULD, and coincidence time window) so as to minimize the contribution of gamma-prompt coincidences. Mouse and rat phantoms were also filled with ^18^F to compare projection profiles on sinograms. This comparison was assessed with phantoms filled with 30 MBq of ^124^I or ^18^F, with a large energy window (250 to 750 keV) and a 4.7-ns coincidence time window.

### Image-quality and activity quantification

**Figure 1:**
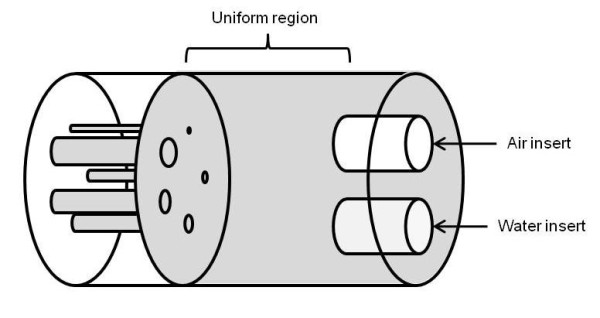
**Schema of the NEMA NU 4-2008 image quality phantom**.

The image-quality phantom described in the NEMA NU 4-2008 procedure is a cylindrical phantom with a 30-mm internal diameter, a 50-mm length, and an internal volume of 20.8 mL. The phantom is divided in three parts (Figure 1), and just the two regions described below were used for image-quality and activity quantification assessment.

The middle part consists of a 20-mm-long hollow cylinder to evaluate the uniformity. A volume of interest [VOI] with a 22.5-mm diameter and 10-mm height was drawn over the center of the uniform region using ImageJ [15]. The average activity concentration [Au_ave_] and percentage standard deviation [%SDu] were calculated within that VOI.

The front part of the phantom consists of two cylinders (8 mm in internal diameter and 15 mm in length). One insert was filled with cold water, and the other, with air. A VOI (4 mm in diameter and 7.5 mm in length) was drawn over each insert. The mean activity (for water and air regions [Aw_ave _and Aa_ave_, respectively]) was determined for the VOI. The spillover ratios for the water and air regions [SOR_w _and SOR_a_, respectively] were calculated as the ratio of Aw_ave_/Au_ave _and Aa_ave_/Au_ave_, respectively.

The phantom was filled with 3 MBq of ^124^I then with ^18^F. Data were acquired for 20 min with the energy window optimized for the mouse phantom (as phantom dimensions fall within the range of a mouse phantom) for ^124^I, and with a 250 to 750 keV energy window for ^18^F. This energy window and a 4.7-ns coincidence time window were chosen for ^18^F after NECR evaluation (Table 1). The list-mode files were rebinned into true coincidence 3D sinograms. Images were reconstructed into a 256 × 256 × 159 matrix (0.388 × 0.388 × 0.796-mm^3 ^pixel size) with FORE-2DFBP using a ramp filter cutoff at the Nyquist frequency and FORE-2DOSEM with 4 iterations (16 subsets). The 3DOSEM/MAP algorithm was also used with 2 iterations (12 subsets) for 3DOSEM followed by 18 iterations of MAP with a request resolution of 1.635 mm (equivalent to a beta parameter of 0.1). Four images were reconstructed for each algorithm: without correction, with attenuation correction alone [AC], with attenuation and scatter corrections [AC-SC], and with attenuation, scatter, and gamma-prompt corrections [AC-SC-GC]. The gamma-prompt correction is not yet available in the IAW software; thus, we applied our correction. Gamma-prompt events were corrected by a weighted (60%, 70%, 80%, 90%, and 100%) uniform subtraction [16]. The 10 outermost projection bins for a projection were averaged. The weighted mean value was then subtracted for each bin value of the projection. Pixels with negative values were set to zero. The corrected sinograms were reconstructed with attenuation and scatter corrections. A specific normalization file was calculated for each set of acquisition parameters using the component-based algorithm as this was strongly recommended by the manufacturer. Images were then calibrated by the manufacturer procedure to convert counts per second in becquerels per milliliter. Calibration factors were determined on an image of a uniform cylinder (diameter of 3 cm and length of 10 cm) filled with a known activity of ^124^I and ^18^F (10 MBq). An acquisition was performed for each energy window for 30 min, and images were reconstructed with algorithms evaluated previously and corrected for attenuation and scatter events. Ten circular regions of interest (diameter of 2 cm) were drawn along the uniform cylinder images, and the mean value of counts per second was calculated. The ASIPro software (Siemens Medical Solutions USA Inc., Knoxville, TN, USA) provided by the manufacturer was used to calculate a calibration factor to convert this value in becquerels per milliliter. This factor takes into account the decay and dead time corrections, the branching ratio, and the reconstruction algorithm.

**Table 1:** NECR value for mouse phantoms filled with 3 MBq of ^18^F

Phantom	Energy window (keV)	Coincidence time window (ns)	NECR (kcps)
Mouse	250 to 750	2.8	140
		4.7	150
	350 to 650	2.8	120
		4.7	130

NECR, noise equivalent count rate.

## Results

### Optimized energy window

**Figure 2:**
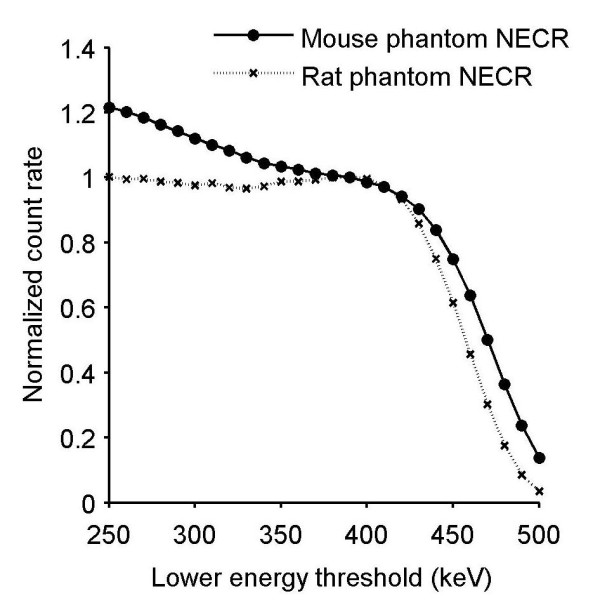
**NECR variation with LLD for the rat (ULD = 550 keV) and mouse (ULD = 590 keV) phantoms**.

The maximum of the NECR was found for an ULD of 550 keV and a LLD of 390 keV for the rat phantom configuration. The optimized ULD was determined to be 590 keV for the mouse phantom, but the NECR increased continuously as the LLD was decreased (Figure 2). As a consequence, two energy windows (a narrow window of 400 to 590 keV and a broad window of 250 to 590 keV) could be selected for the mouse phantom. Therefore, it was necessary to study other figures of merit as scatter and gamma-prompt fractions to choose the appropriate energy window.

### Noise equivalent count rate, scatter fraction, and gamma-prompt fractions

**Figure 3:**
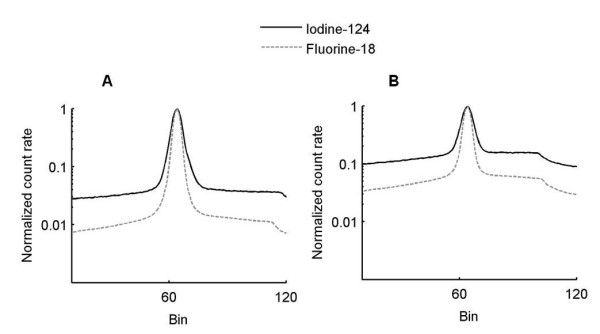
**Profiles drawn on summed sinograms for ^124^I and ^18^F filled mouse-sized (A) and rat-sized (B) phantoms**.

**Figure 4:**
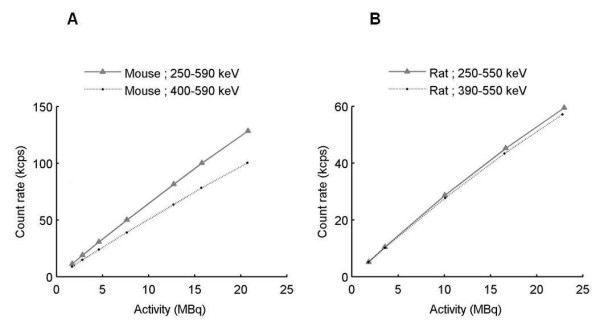
**NECR curve for mouse-sized (A) and rat-sized (B) phantoms filled with ^124^I**.

**Figure 5:**
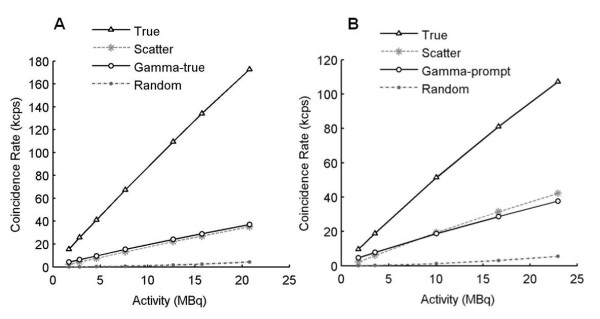
**Coincidence rates for (A) mouse-sized (250 to 590 keV) and (B) rat-sized (250 to 550 keV) phantoms**.

Figure 3 shows the profiles drawn on the summed sinograms for the rat and mouse phantoms filled with ^124^I and ^18^F. The difference between the isotope distributions confirms the choice of a uniform correction for gamma-prompt events. The NECR curves acquired for the mouse and rat phantoms were not affected by the variation of the coincidence time window. NECR curves were calculated for different energy windows to complete (for the mouse phantom) and confirm (for the rat phantom) previous results found by varying the LLD and ULD. The NECR measured with 400 to 590 keV and 250 to 590 keV energy windows for the mouse phantom, and 390 to 550 keV and 250 to 550 keV energy windows for the rat phantom are displayed in Figure 4a, b. Figure 5a, b shows coincidence rates for the mouse (250 to 590 keV) and rat (250 to 550 keV) phantoms. The NECR was decreased with the use of a higher LLD (400 keV instead of 250 keV) for the mouse phantom with a ULD fixed at 590 keV. The NECR did not present a significant variation when the LLD was increased for the rat phantom. The scatter fraction and the gamma-prompt fraction for the mouse and rat phantoms are shown in Table 2 for all energy windows and with a 4.7-ns coincidence time window. For the mouse and rat phantoms, GF decreases with increasing LLD. Then, the ratio between gamma-prompt and scatter coincidences was the same for the mouse and rat phantoms for comparable energy windows (1.15 for 250 to 590 keV and 250 to 550 keV, respectively). Energy windows of 250 to 590 keV and 250 to 550 keV were more suitable for the mouse and rat phantoms based on the NECR curve. However, the scatter fraction and the gamma-prompt fraction decrease with a narrower window (400 to 590 keV for the mouse phantom and 390 to 550 keV for the rat phantom); thus, they were chosen for the image-quality and activity quantification assessment.

**Table 2:** SF and GF for the mouse and rat phantoms

Phantom	Energy window (keV)	SF (%)	GF (%)
Rat	250 to 550	25.4	29.2
	390 to 550	19.3	10.3
Mouse	250 to 590	16.6	19.1
	400 to 590	13.7	10.2

SF, scatter coincidence fraction; GF, gamma-prompt coincidence fraction.

### Image-quality and activity quantification

The %SDu in the uniform region and the bias between the measured Au_ave _and injected activity calculated for ^124^I and ^18^F are shown in Table 3, respectively, for 400 to 590 keV and 250 to 750 keV energy windows and a 4.7-ns coincidence time window. Results were presented for various reconstruction algorithms and with or without attenuation, scatter, and gamma-prompt corrections. Only the 100% weighted uniform subtraction was reported because no difference was found between 60% and 100% of a uniform distribution subtraction. The measured Au_ave _was underestimated by 9.9% for a 3DOSEM/MAP reconstruction with a phantom filled with ^124^I. This algorithm presents the lower bias on the quantification. When ^18^F-based acquisitions were considered, the Au_ave _was recovered with a better accuracy (3.7% for 3DOSEM/MAP reconstruction) than for ^124^I-based acquisition. For the three reconstruction algorithms, attenuation correction applied alone improved the value of Au_ave _(for example, the bias on measured Au_ave _for 3DOSEM/MAP varied from -34.3% to -3.6%), but scatter correction degraded the activity quantification (from -3.6% to -9.9% in the same example). The gamma-prompt correction increases underestimation (-23% for 3DOSEM/MAP reconstruction). However, both attenuation and scatter corrections do not have an important impact on the %SDu, which are comparable for all reconstructions. The spillover ratios (SOR_w _and SOR_a_) are presented in Table 4. Attenuation and scatter corrections decrease SOR_a _for each algorithm and for both isotopes. On the other hand, attenuation correction applied alone increased SOR_w _(from 3.6 to 8.9 for 3DOSEM/MAP algorithm for ^124^I). This effect was more important for ^124^I than for ^18^F. Scatter correction and gamma-prompt corrections decreased SOR_w_. Finally, SOR_a _and SOR_w _were close to zero with the application of all corrections on ^124^I-based acquisition and particularly for the 3DOSEM/MAP reconstruction.

**Table 3:** %SDu and the relative error between average activity measured and injected activity

Isotope	Correction	%SDu			Error (%)		
		2DFBP	2DOSEM	3DOSEM/MAP	2DFBP	2DOSEM	3DOSEM/MAP
^18^F	No correction	6.3	6.7	6.2	-28.3	-28.4	-25.2
	AC	5.0	5.5	5.8	0.6	0.5	6.2
	AC-SC	5.1	5.7	4.8	-1.7	-2.1	3.7
^124^I	No correction	14.3	16.6	15.6	-36.2	-34.7	-34.3
	AC	13.8	16.3	14.5	-8.0	-6.9	-3.6
	AC-SC	14.4	17.8	15.2	-13.7	-13.4	-9.9
	AC-SC-GC	16.9	21.3	14.2	-27.2	-26.6	-23.0

%SDU, percentage standard deviation; 2DFBP, 2D filtered back-projection; 2DOSEM, 2D ordered-subset expectation maximization; 3DOSEM/MAP, 3DOSEM in combination with maximum *a posteriori*; AC, attenuation correction; SC, scatter correction; GC, gamma-coincidence correction.

**Table 4:** SORa and SORw for the image quality phantom

Isotope	Correction	SOR_a _(%)			SOR_w _(%)		
		2DFBP	2DOSEM	3DOSEM/MAP	2DFBP	2DOSEM	3DOSEM/MAP
^18^F	No correction	15.9	15.8	19.9	-0.8	4.4	0.1
	AC	2.6	5.8	1.2	2.4	5.6	3.3
	AC-SC	1.4	4.9	3.0	0.2	4.2	2.8
^124^I	No correction	17.3	18.1	17.9	4.8	9.7	3.6
	AC	3.2	7.0	< 0.01	8.5	12.5	8.9
	AC-SC	-0.9	4.5	< 0.01	3.7	7.4	3.6
	AC-SC-GC	-6.6	2.2	< 0.01	-4.4	2.9	0.9

SOR_a_, spillover ratio for the air region; SOR_w_, spillover ratio for the water region; 2DFBP, 2D filtered back-projection; 2DOSEM, 2D ordered-subset expectation maximization; 3DOSEM/MAP, 3DOSEM in combination with maximum *a posteriori*; AC, attenuation correction; SC, scatter correction; GC, gamma-coincidence correction.

## Discussion

The emission characteristics of ^124^I imply that an optimization of acquisition parameters is required. To date, the challenges of ^124^I PET imaging have mainly been analyzed for clinical applications [8-10,14], and most approaches have consisted of Monte Carlo modeling of the detector [17,18]. The aim of this work was a first step to define the best experimental acquisition settings for mouse and rat phantoms for the Siemens Inveon PET system, based on the NECR figure of merit. In a second part, algorithms for reconstruction and corrections available on the Inveon Acquisition Workplace 1.5 were assessed with the image-quality and activity quantification evaluation.

### Acquisition setting optimization for the mouse and rat phantoms

The probability of detecting a single photon in coincidence with a 511-keV photon was high for the Inveon PET system given the long, axial FOV and a large acceptance angle of 38.3° [19]. The easiest way to remove most of the spurious coincidences is to optimize the acquisition energy windows, and the NECR determination is the figure of merit commonly used [12,14,17,20,21]. The optimized energy window thresholds are found when the NECR reaches its maximum. The optimized ULD was 590 keV for the mouse-sized phantoms, but the lack of a maximum NECR when varying the LLD led us to choose two energy windows (250 to 590 keV and 400 to 590 keV) for further analysis. The optimized ULD was 550 keV for the rat, and the LLD was 390 keV. This value was in agreement with the simulation study of the Inveon system for ^124^I [18]. The NECR for the mouse phantom was more affected by the adjustment of the LLD than that for the rat phantom. No difference was found for NECR curves acquired with a 2.8- or 4.7-ns coincidence time window for both phantoms and for the activity range used in preclinical imaging. GF was identical for rat and mouse phantoms for a narrow energy window (390 to 550 keV and 400 to 590 keV, respectively), but GF was higher for the rat (29.2%) than for the mouse (19.1%) for a larger energy window (250 to 550 keV and 250 to 590 keV, respectively). GF evaluation with a uniform distribution based on the tail distribution was not optimal because scatter events can be counted as gamma-prompt events. When the GF was calculated on additional ^18^F measurements (data not shown), a value of 3% was found for rat and mouse phantoms with 250 to 750 keV and 4.7-ns acquisition windows. Part of these gamma-prompt events corresponds to scattered events due to interactions in the gantry [22]. Consequently, a uniform evaluation of gamma-prompt events overestimated the GF. Looking at the NECR, SF, and GF, an optimum setting was chosen, 400 to 590 keV for the mouse phantom and 390 to 550 keV for the rat phantom.

### Reconstruction algorithm and correction evaluation for the mouse phantom

Application of scatter correction on a uniform region of the image-quality phantom (mouse-sized phantom) confirmed results presented elsewhere [22,23]. SSS correction overestimates scatter coincidences for all isotopes, particularly in small objects. Indeed, the estimation of scattered events based on tail distributions also include scatter in the detector and gantry. Furthermore, gamma-prompt events outside the phantom were considered as object-scattered events, which increased the error on scattered event estimation with SSS correction. Activity quantification in a cold air region was improved by attenuation correction (lower spillover ratio), while it was degraded in a cold water region (higher spillover ratio) for both isotopes (Table 4), albeit less significantly for ^18^F. These results bring into question the CT-based attenuation correction implemented in the system and particularly for non-pure isotopes. In fact, 603-keV photons were considered as 511-keV photons, and corresponding attenuation coefficients were overestimated. However, the improvement obtained on ^18^F activity quantification in the uniform region with attenuation correction shows that attenuation should be corrected for small animals [24]. Scatter correction added to attenuation correction improved the quantification for air and water cold inserts for the three reconstruction algorithms. Conclusions about scatter and attenuation corrections on activity quantification should be considered with caution. In fact, the reference used to evaluate these corrections was the activity injected as defined by the dose calibrator. The uncertainty of 11% on that value was, on average, higher than the deviations observed on activity quantification in the uniform region, SOR_a _and SOR_w_, when the attenuation or scatter corrections were applied. Thus, the low background measures in the cold insert could be caused by reconstruction noise and by the positron range, particularly in the water cold insert [25]. With a mean range of 3.5 mm in water, positrons emitted around the cold insert could be annihilated inside it with an electron of water. Under this condition, a gamma-prompt correction does not seem to be necessary even if it decreases the background in the cold region. In addition, this correction deletes information in the hot region with an important degradation of the quantification. Moreover, no difference was found between the corrections with 100% and 60% uniform distribution subtraction in this small animal context compared to clinical imaging [16]. Correction of gamma-prompt events does not seem to be essential for mouse imaging with ^124^I because of the low gamma-prompt background in the sinogram file for acquisitions with an optimized energy window. Refined approaches such as a deconvolution method [26] or analytical modeling [27,28] could bring an improvement in the activity quantification compared to uniform distribution subtraction.

Energy windows of 400 to 590 keV and 390 to 550 keV are recommended for ^124^I mouse and rat studies, respectively, with the Inveon system. These energy window settings exclude part of the scattered coincidences and gamma-prompt events. The 3DOSEM/MAP algorithm presents the most accurate activity quantification with attenuation and scatter corrections.

## Conclusions

This work determined the optimal acquisition parameters for ^124^I in preclinical imaging with the Inveon PET system. The physical characteristics of ^124^I did not impact on imaging performance equally for mouse- and rat-sized objects, partly because of differences in attenuation and scattered volumes. We derived an optimized combination of acquisition settings for both mouse and rat phantoms. These parameters will now be applied in molecular imaging preclinical experiments performed in our laboratory. Quantitative imaging results will be compared to experimental *ex vivo *counting, in order to determine the accuracy of quantitative imaging, both for ^18^F and ^124^I PET preclinical imaging. In parallel, modeling of the Inveon PET system is ongoing in our laboratory [29] in order to better study the impact of the ^124^I emission spectrum on the detection process.

## Competing interests

The authors declare that they have no competing interests.

## Authors' contributions

NA carried out the acquisition and the image analyses. TC participated in the analyses. CH was responsible of the PET/CT system. MB conceived the study and participated in its design and coordination. JB participated in the study coordination. All authors read and approved the final manuscript.
